# Type II Collagen from Cartilage of *Acipenser baerii* Promotes Wound Healing in Human Dermal Fibroblasts and in Mouse Skin

**DOI:** 10.3390/md18100511

**Published:** 2020-10-11

**Authors:** Ching-Shu Lai, Chun-Wei Tu, Hsing-Chun Kuo, Pei-Pei Sun, Mei-Ling Tsai

**Affiliations:** 1 Department of Seafood Science, National Kaohsiung University of Science and Technology, Kaohsiung 811, Taiwan; chlai@nkust.edu.tw (C.-S.L.); 1061534108@nkust.edu.tw (C.-W.T.); ppsun@nkust.edu.tw (P.-P.S.); 2 Department of Nursing, Division of Basic Medical Sciences, Chang Gung University of Science and Technology, Chiayi 61363, Taiwan; guscsi@gmail.com

**Keywords:** type II collagen, sturgeon cartilage, by-product, wound healing, fibroblast

## Abstract

Type II collagen is an important component of cartilage; however, little is known about its effect on skin wound healing. In this study, type II collagen was extracted from the cartilage of *Acipenser baerii* and its effect on in vitro and in vivo wound healing was compared to type I collagen derived from tilapia skin. Sturgeon cartilage collagen (SCC) was composed of α1 chains and with a thermal denaturation (T_d_) at 22.5 and melting temperature (T_m_) at 72.5 °C. Coating SCC potentiated proliferation, migration, and invasion of human dermal fibroblast adult (HDFa) cells. Furthermore, SCC upregulated the gene expression of extracellular matrix (ECM) components (col Iα1, col IIIα1, elastin, and Has2) and epithelial-mesenchymal transition (EMT) molecules (N-cadherin, Snail, and MMP-1) in HDFa. Pretreatment with Akt and mitogen-activated protein kinase (MAPK) inhibitors significantly attenuated the HDFa invasion caused by SCC. In mice, the application of SCC on dorsal wounds effectively facilitated wound healing as evidenced by 40–59% wound contraction, whereas the untreated wounds were 18%. We observed that SCC reduced inflammation, promoted granulation, tissue formation, and ECM deposition, as well as re-epithelialization in skin wounds. In addition, SCC markedly upregulated the production of growth factors in the dermis, and dermal and subcutaneous white adipose tissue; in contrast, the administration of tilapia skin collagen (TSC) characterized by typical type I collagen was mainly expressed in the epidermis. Collectively, these findings indicate SCC accelerated wound healing by targeting fibroblast in vitro and in vivo.

## 1. Introduction

The skin is the largest organ in mammalian organisms. It acts as the main barrier and plays several vital roles in immunity, fluid homeostasis, thermoregulation, excretion, sensation, and metabolic functions of the body [1]. When the skin is wounded, the tissue undergoes healing in the ordered steps of homeostasis, inflammation, proliferation, and remodeling, to promote wound closure and eventually restore the normal tissues [1,2]. This physiological healing process is highly coordinated and involves interactions between various cellular and molecular networks in the wound [2,3]. In general, the acute wound usually heals completely within weeks with normal healing phases and has normal wound physiology. However, chronic wounds are characterized by a persistent inflammatory phase, which are difficult to heal within months or a year [4]. Moreover, chronic wounds are medical and healthcare burdens that result in a poor quality of life in patients, causing pain, loss of mobility, and increased mortality. Therefore, effective medical strategies and appropriate care are crucial for the management of skin wounds and the improvement of wound healing.

To facilitate wound healing and also prevent microbial infection, a number of dressing products for wound treatment and care have been developed by targeting different aspects of the healing process [5]. In recent years, the use of biological materials as wound dressings has gained momentum, including the use of polysaccharides (chitosan, alginate, fucoidan, etc.) and proteins (keratin, fibrin, collagen, etc.) owing to their biocompatibility, biodegradability, and non-toxic nature [6]. Collagens are the most abundant structural proteins, existing as a fibril with the basic triple helical structure in the skin, bone, and other connective tissue in mammals. Among the 28 members of collagen, type I collagen is the most abundant in the body. It demonstrates excellent physical and mechanical properties and is considered as a unique dressing material in wound treatment and care [7].

Fish collagen is produced from by-products of processing including skin, scales, fins, and bone, which display various biological properties in skin wound healing, engineering, and regeneration [8,9]. Collagen from the tilapia facilitates skin regeneration and wound healing by stimulating adhesion, proliferation, migration, angiogenesis, and epidermal differentiation in keratinocytes, fibroblasts, and wounded animals [10,11,12]. This is type I collagen and is comparable to that derived from terrestrial sources, despite being the predominant collagen in skin and scales. Other common types such as types II, III, and IV are distributed in other tissues of fish and may present different beneficial and biochemical properties in wound healing. Type II collagen is also a fibril-forming collagen, with a different polypeptide chain composition compared to type I collagen [13]. As the main component of cartilage, type II collagen is important for providing tensile strength to the tissue [14]. Type II collagen from marine materials such as tilapia scale, chum salmon, Rohu, Catla, shark skin, and cartilage has been used as a biomaterial or in combination with other functional scaffolds that are widely applied to bone and cartilage tissue engineering and regeneration [9,15]; however, it is less known for its effect on skin wound healing. Sturgeon is one of the most valuable fish worldwide, serving as an important source of meat and caviar, and with considerable processing wastes including the cartilage [16]. We extracted and identified type II collagen from the cartilage of a sturgeon, *Acipenser baerii*, the biological effect of which had not been reported. Here, we aimed to investigate the effect of the collagen extracted from *Acipenser baerii* on wound healing in vitro and in vivo. We also included tilapia skin collagen (TSC) as a positive control for the wound healing effect, because it is typical of type I collagen and its wound healing properties are well documented. We demonstrated that collagen from *Acipenser baerii* and tilapia skin collagen (TSC) both stimulated human fibroblast proliferation, migration, and wound healing in mice, but with different targeted cell types in the skin tissue.

## 2. Results

### 2.1. Characterization of Sturgeon Cartilage Collagen (SCC)

The proximate composition of cartilage from *Acipenser baerii* revealed protein as the major component (Table S1). The yield of SCC from cartilage was 20.11 ± 0.44% based on the lyophilized weight of the initial samples (Table S2). The collagen content of cartilage from *Acipenser baerii* was about 28%, comparable to other sturgeons as reported by Liang et al. [17] and Luo et al. [18]. The SDS-PAGE demonstrated that the pattern of SCC was similar to the type II collagen from the cartilage of Amur sturgeon and Siberian sturgeon [17,18]. In contrast, TSC consists of two different α chains (α1 and α2) and a high molecular weight α chain (Figure S1A) similar to that in rat type I collagen as reported by Li et al. [19]. A scanning electron microscope (SEM) was performed to investigate the surface morphology and porosity of lyophilized SCC. As shown in Figure S1B, SCC displayed an irregular thick sheet-like structure attached by random-coiled fibers, comparable to the collagen from the cartilage of Amur sturgeon and Siberian sturgeon [17,18]. The amino acid profile (Table S3) showed glycine (Gly) was the most abundant amino acid in both SCC and TSC, followed by proline (Pro) and hydroxyproline (Hyp). The content of Gly, Pro, and Hyp in SCC and TSC was similar, but the content of Ala was slightly higher in TSC than in SCC. The amino acid content of SCC was also similar to TSC. The thermal denaturation (Td) of SCC was 22.5 °C and the melting temperature (Tm) was 72.5 °C (Figure S1C). The Td and Tm were lower than that observed in cartilage collagen from Amur sturgeon and Siberian sturgeon [17,18] and could be attributed to the difference in fish species, cultivating environment, as well as the amino acid composition and covalent crosslinks of collagen [20,21].

### 2.2. SCC Increased Fibroblasts and Keratinocytes Proliferation

**Figure 1:**
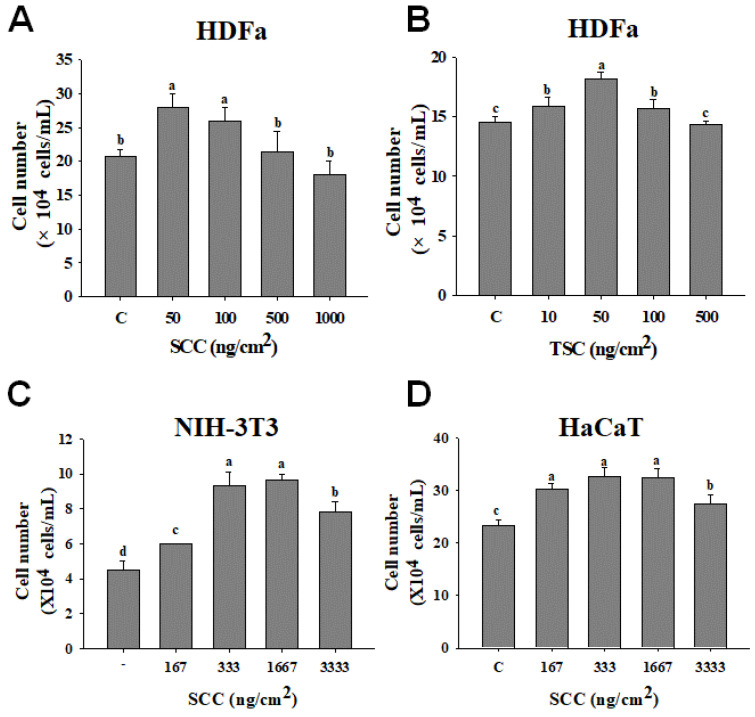
Effect of sturgeon cartilage collagen (SCC) on proliferation of human dermal fibroblasts, keratinocytes, and dermal fibroblasts. The 24-well plates were coated with SCC or tilapia skin collagen (TSC) overnight and (**A**,**B**) human dermal fibroblast adult (HDFa), (**C**) NIH-3T3, and (**D**) HaCaT cells were seeded on coating wells for 36 h. Cell were then collected and the cell number was measured by the trypan blue assay. Mean values within each column with different labels (a–d) are significantly different (*p* < 0.05) according to one-way ANOVA analysis of variance and Duncan’s multiple range test.

Dermal fibroblasts are critical in tissue repair and remodeling [22]. The trypan blue assay was used to investigate the effect of coating SCC on cell number of human fibroblasts. As shown in Figure 1A, coating 50 and 100 ng/cm^2^ SCC significantly increased the proliferation of human dermal fibroblast adult (HDFa) cells from 100% (control group) to 136 and 126%, respectively. Furthermore, coating TSC also induced the proliferation of HDFa from 98 to 126% (compared to the control group), with the maximal increase observed at a concentration of 50 ng/cm^2^ (Figure 1B). Notably, SCC and TSC displayed no cytotoxic effect of HDFa, with >95% cell viability by coating at various concentrations (data not shown). This result suggested that coating SCC increased the proliferation of human fibroblasts. We also investigated the effect of coating SCC on NIH-3T3 embryonic mouse skin fibroblasts, another common cell line used in in vitro wound healing model [23]. We found coating SCC markedly induced the proliferation of NIH-3T3 fibroblasts from 133 to 216% (Figure 1C). Epidermal keratinocytes are also important in the wound healing process [24]. To further examine the effect of coating SCC on epidermal keratinocytes proliferation, the immortalized human keratinocyte cell line HaCaT was used [25]. Figure 1D showed the SCC coating induced a marked increase in cell numbers in HaCaT cells. 

### 2.3. SCC Promoted Migration and Invasion on HDFa

**Figure 2:**
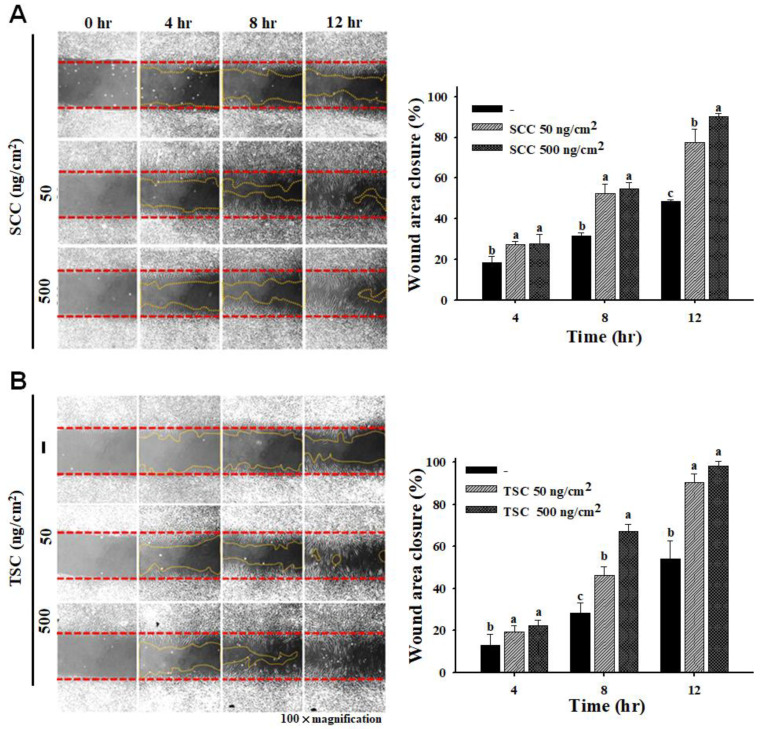
Effect of coating SCC on cell migration of HDFa. HDFa were cultured on TSC-coated or SCC-coated wells for 12 h and cell migration was measured by the scratching assay. (**A**) The images of cell migration in (**A**) SCC- or (**B**) TSC-coated groups were taken immediately after scratching at 0, 4, 8, and 12 h (magnification, × 100). The wound healing area were measured. Mean values within each column with different labels (a–c) are significantly different (*p* < 0.05) according to one-way ANOVA analysis of variance and Duncan’s multiple range test.

**Figure 3:**
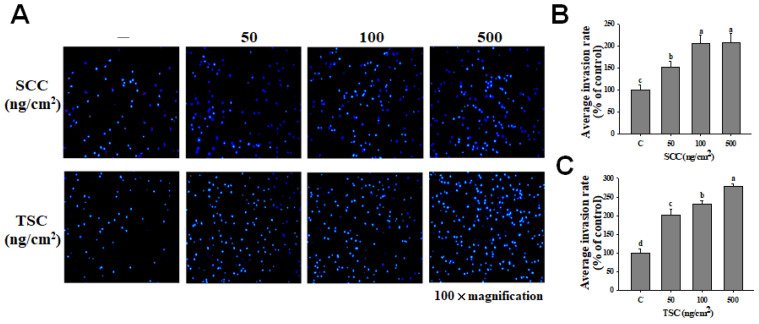
Effect of coating SCC on cell invasion of HDFa. SCC or TSC coating is described in Materials and Methods. (**A**) HDFa cells were cultured on SCC-coated or TSC-coated transwell for 16 h, and the invaded cells were stained with 4′,6-diamidino-2-phenylindole (DAPI). (**B**,**C**) A representative number of invading cells through the membrane were counted under the microscope in four random fields at a 100× magnification. Mean values within each column with different labels (a–c) are significantly different (*p* < 0.05) according to one-way ANOVA analysis of variance and Duncan’s multiple range test.

**Figure 4:**
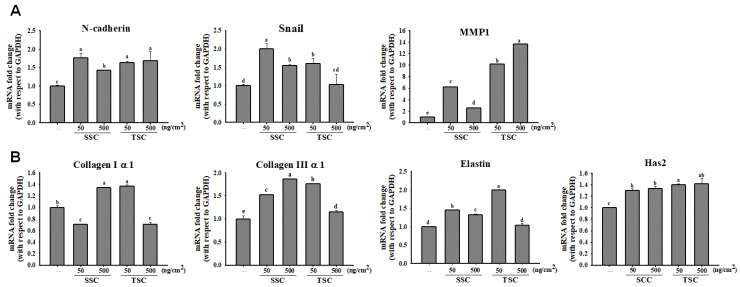
Effect of coating SCC on gene expression of cell migration and extracellular matrix (ECM) markers in HDFa. HDFa were cultured on TSC- or SCC-coated dishes for indicated times. The RNA was extracted and qRT-PCR analysis was carried out to determine the gene expressions of (**A**) migration markers (N-cadherin, Snail, and MMP 1) at 6 h and (**B**) ECM markers (collagen Iα1, collagen IIIα1, elastin, and hyaluronan synthases 2) at day 2. Mean values within each column with different labels (a–e) are significantly different (*p* < 0.05) according to one-way ANOVA analysis of variance and Duncan’s multiple range test.

We examined the effect of SCC on promoting the migration of fibroblasts by using a scratch wound assay. Coating 50 and 500 ng/cm^2^ of SCC significantly induced HDFa migration, resulting in wound closure in a time-dependent manner (Figure 2A). The percentage of wound closure by coating 50 and 500 ng/cm^2^ of SCC at 12 h was 77.6 and 90.3%, respectively, compared to 48.3% in the uncoated group. Coating 50 and 500 ng/cm^2^ TSC also caused 90.2 and 98% wound closures, respectively, compared to 54.1% in the uncoated group at 12 h (Figure 2B). Figure 3A displays a significant and dose-dependent increase in the number of invaded cells in the coating SCC group. The invasion rates of 50, 100, and 500 ng/cm^2^ SCC were 152, 206, and 207%, respectively. Coating 50, 100, and 500 ng/cm^2^ TSC showed the invasion rates at 147, 157, and 258%, respectively (Figure 3B,C). The above results demonstrated that coating SCC or TSC promoted the migration and invasion of human fibroblasts. Moreover, the gene expressions of mesenchymal markers, N-cadherin, Snail [26], and matrix metalloproteinase-1 (MMP-1) were upregulated by both SCC and TSC coating (Figure 4A). Fibroblasts are the major cells to synthetize the extracellular matrix (ECM) protein during wound healing [27]. We showed upregulated gene expressions of collagen Iα1, collagen IIIα1, elastin, and Has2 were observed in the SCC group (Figure 4B), suggesting the ability of coating SCC to stimulate ECM production in fibroblasts.

### 2.4. SCC Promoted Migration and Invasion via Regulation of Mitogen-Activated Protein Kinases (MAPKs)

**Figure 5:**
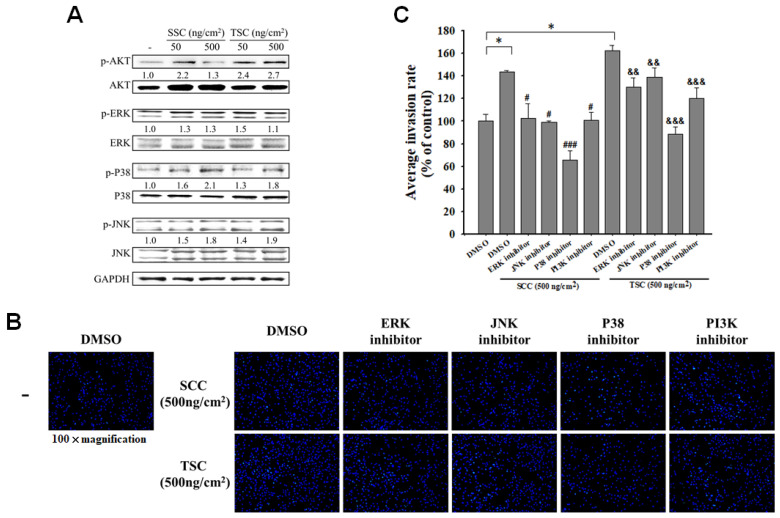
Effect of coating SCC on mitogen-activated protein kinases (MAPKs) and Akt signaling in HDFa. (**A**) HDFa cells were cultured on SCC- or TSC-coated dishes for 24 h. Total protein was extracted and the protein expressions of p-AKT/AKT, p-ERK/ERK, p-P38/P38, and p-JNK/JNK were analyzed by western blot. The relative protein is expressed as the fold increased in comparison to uncoated group after normalized to glyceraldehyde 3-phosphate dehydrogenase (GAPDH). (**B**) HDFa cells were cultured on TSC-coated transwell for 1 h, and treated with 10 mmoL PD98059 (inhibitor of Erk), 10 mmoL SP600125 (inhibitor of JNK), 10 mmoL SB203580 (inhibitor of p38), and 10 mmoL KT5720 (inhibitor of Akt) for another 15 h, and the invaded cells were stained with DAPI. (**C**) The number of invading cells was counted under the microscope in four random fields at a 100× magnification. The results are expressed as mean ± SD, * *p* < 0.05 vs. DMSO alone; ^#^ *p* < 0.05, ^##^ *p* < 0.01, and ^###^ *p* < 0.001 vs. SCC-coated HDFa treated with DMSO; ^&^ *p* < 0.05, ^&&^ *p* < 0.01, and ^&&&^ *p* < 0.001 vs. TSC-coated HDFa treated with DMSO. Statistical significance of two groups was determined using Student’s *t*-test.

The phosphoinositide 3 kinase (PI3K)/Akt and mitogen-activated protein kinases (MAPKs), including Jun N-terminus kinase (JNK), p38, and ERK, play crucial roles in cell migration by modulation of their downstream signaling molecules, proteins, and enzymes [28,29]. Figure 5A showed the phosphorylation of Akt and MAPKs were found in HDFa with coating SCC. To examine whether the promoting effect of coating SCC on fibroblast migration and invasion was Akt- and MAPK-dependent, individual inhibitors were used. The results of the transwell assay demonstrated that coating SCC promoted migration of HDFa cells, but this was significantly decreased by treatment with Akt and MAPKs inhibitors (Figure 5B). Inhibition of p38 drastically abolished SCC-induced invasion of HDFa (Figure 5C). The above effects were also found in TSC coating. These results revealed that coating SCC or TSC promoted the invasion of HDFa via the Akt- and MAPK-dependent mechanism.

### 2.5. Topical Application of SCC Accelerated Skin Wound Healing

**Figure 6:**
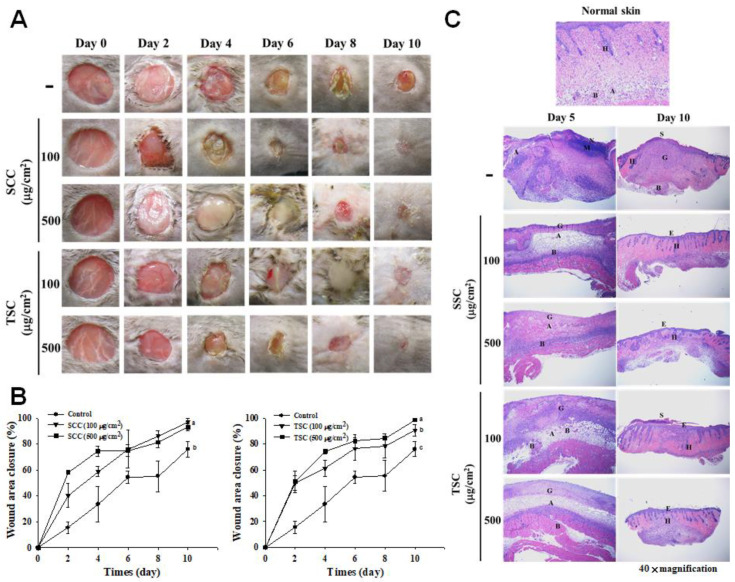
Effect of SCC on promoting wound healing in C57BL/6J mice. SCC or TSC application on skin wound was described as Materials and Methods. Wound closure is expressed as a percentage of initial wound area at day 0. (**A**) Representative photographs of the wounds at days 0, 2, 4, 6, 8, and 10 after creating skin excisional wounds. (**B**) Quantitative analysis of SCC or TSC improved the rate of wound closure. Mean values within each column with different labels (a–c) are significantly different (*p* < 0.05) according to one-way ANOVA analysis of variance and Duncan’s multiple range test. (**C**) Histopathology of skin wounds at day 5 and day 10 after surgery. S: Scab; G: Granulation; E: Epidermis; A: Adipocyte; H: Hair follicle; B: Blood vessel; N: Necrosis; M: Inflammatory cells.

We further investigated whether SCC promotes skin wound healing in vivo. Figure 6A illustrates that SCC application significantly accelerated skin wound healing as compared to untreated wounds during the experiment. The percentage of wound area closure in the untreated wounds was 18%, whereas 100 and 500 μg/cm^2^ SCC or TSC treatments showed wound closures of 40 and 59, 50, and 50%, respectively, on day 2 (Figure 6B). The skin wounds were almost closed completely in SCC- or TSC-treated groups, while the untreated group showed a visible skin wound at day 10. Histological analysis showed normal skin displaying the epidermis, the dermal layer with clear hair follicle, and the subcutaneous layer rich in adipocytes (Figure 6C). The untreated mice revealed skin disorganization of the wound area, followed by a massive inflammatory infiltration, and necrotic tissue at day 5, demonstrating an inflammation phase of wound healing. On day 10, the wound area in untreated mice was covered by a scab and a neoepithelium was visible under the scab, followed by the granulation tissue formation, suggesting that the healing process was progressing to the proliferative phase. In contrast to the untreated mice, the skin tissue was apparently organized in the wound areas treated with SCC or TSC application at day 5, with less extent of inflammation, appearance of blood vessels, and granulation tissue, indicating accelerated healing kinetics. The wounds were contracted, and the granulation tissue was almost covered by epithelium, with clear angiogenesis and hair follicles in all SCC- or TSC-treated groups on day 10 (Figure 6C, right). 

**Figure 7:**
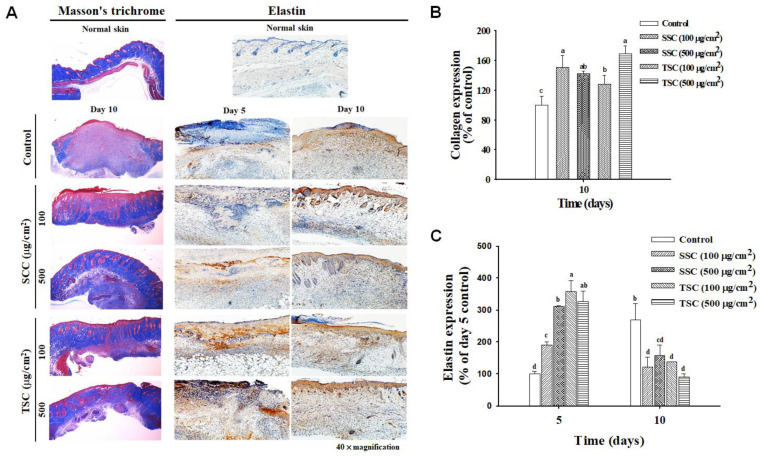
SCC promoted collagen deposition and elastin expression in skin wounds. (**A**) Representative images of Masson’s trichrome staining (left) and immunohistochemistry (IHC) staining for elastin (right) in the skin wound of C57BL/6J mice. The blue color represents the stained collagen in Masson’s trichrome staining and the expression of elastin is shown as brown color by IHC staining. (**B**,**C**) Quantitative analysis of collagen deposition and elastin immunostaining, respectively. Mean values within each column with different labels (a–d) are significantly different (*p* < 0.05) according to one-way ANOVA analysis of variance and Duncan’s multiple range test.

The results of Masson’s trichrome staining showed that collagen (stained with blue color) was abundant and evenly distributed in the epidermal and dermal layer in normal skin but the density of collagen found at the wound of untreated mice was low (Figure 7A, left; Figure 7B). SCC-treated mice presented an organized and abundant collagen deposition compared to the untreated group on day 10. Immunohistochemical staining demonstrated that the level of elastin deposition was significantly higher in the SCC-treated groups on day 5 and day 10 compared to untreated mice (Figure 7A, right; Figure 7C). A similar effect occurred in the TSC-treated group. These results suggested that SCC application promoted ECM deposition and accelerated wound healing in mouse skin.

### 2.6. Upregulated Growth Factors Contributed to SCC-Mediated Acceleration of Wound Healing

**Figure 8:**
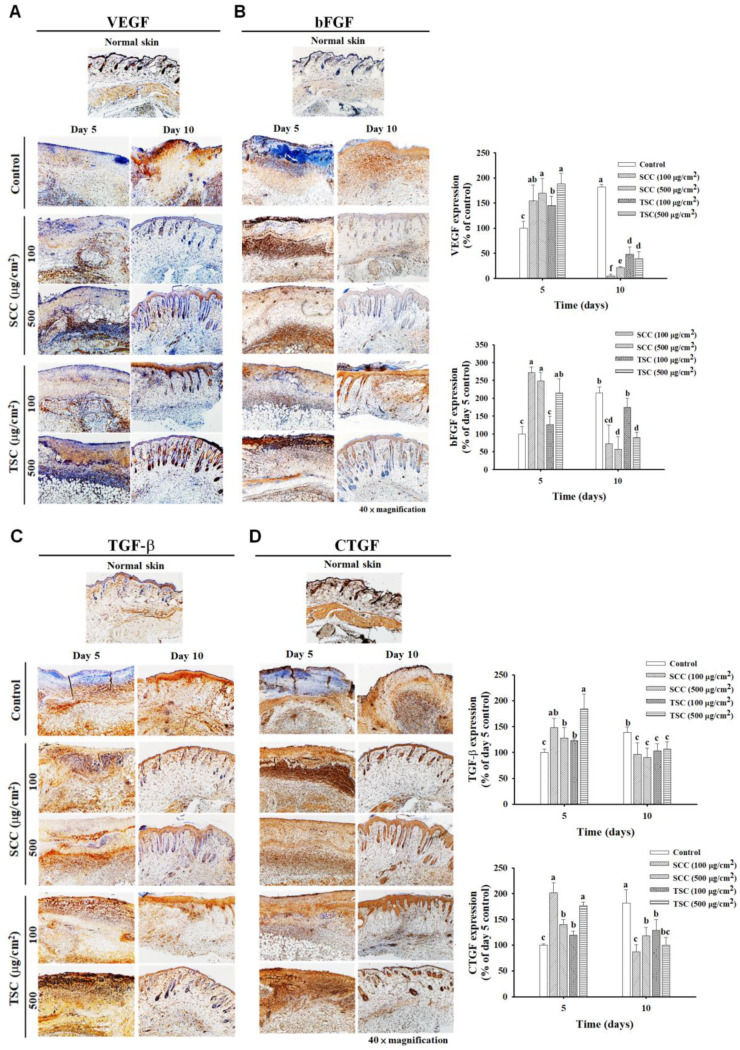
SCC-promoted growth factors secretion in skin wounds. The sections of skin wound from C57BL/6J mice at day 5 and day 10 were stained with (**A**) vascular endothelial growth factor (VEGF), (**B**) basic fibroblast growth factor (bFGF), (**C**) transforming growth factor-β (TGF-β), and (**D**) connective tissue growth factor (CTGF) (brown color) by immunohistochemistry. The relative expression of growth factors was counted in at least five different fields in each mouse and quantified by Image J. Mean values within each column with different labels (a–f) are significantly different (*p* < 0.05) according to one-way ANOVA analysis of variance and Duncan’s multiple range test.

To investigate the potential growth factors involved in SCC-promoted wound healing, immunohistochemistry (IHC) staining was performed to examine vascular endothelial growth factor (VEGF), basic fibroblast growth factor (bFGF), transforming growth factor-β (TGF-β), and connective tissue growth factor (CTGF) expression in the wounds. We observed that VEGF was less expressed in the wound area of untreated mice compared to normal skin at day 5 and was gradually increased at day 10 (Figure 8A). In comparison to untreated mice, the application of SCC significantly increased VEGF expression on day 5 and was reduced on day 10. Upregulated VEGF was also found in TSC treated mice. However, the expression of VEGF was demonstrated in the dermis (fibroblasts), dermal, and subcutaneous white adipose tissue by 500 μg/cm^2^ SCC treatment while TSC application (500 μg/cm^2^) showed elevated VEGF expression in the epidermis (keratinocytes) on day 5. A similar expression pattern of VEGF was observed in normal skin and SCC or TSC groups on day 10, representing a higher level in the epidermal layer of skin. Increased bFGF, TGF-β, and CTGF levels in the wound area were detected in the untreated group compared to normal skin. SCC or TSC administration both displayed enhanced expressions of bFGF, TGF-β, and CTGF compared to the untreated group (Figure 8B–D). Interestingly, the expression pattern of bFGF, TGF-β, and CTGF was similar to VEGF between the SCC- and TSC-treated groups. All these growth factors were highly expressed within the dermis, dermal, and subcutaneous white adipose tissue in SCC-treated groups. In TSC administration, the growth factors were abundantly expressed in the epidermal layer. These results indicated that SCC and TSC might target different cells to produce growth factors that are involved in the acceleration of wound closure.

## 3. Discussion

In the current study, we found that despite SCC sharing a similar amino acid profile to the type II collagen from the cartilage of Amur sturgeon and Siberian sturgeon [17,18], the denaturation temperature of SCC is lower than that of these two sturgeons. This may contribute to the difference in amino acid composition and sequence, the number and the nature of covalent crosslinks, as well as the molecular conformation of type II collagen. In addition, in our study, TSC was identified as being composed of one α1 chain and one α2 chain, similar to the result by Li et al. [19]. The result of amino acid profile showed the content of Pro, Hyp, Gly, Ala, and Lys of TSC was slightly different than that in the reports of Li et al. and Song et al. [19,30]. 

We further investigated the effect of type II collagen extracted from the cartilage of *Acipenser baerii* on wound healing in vitro and in vivo. For the first time, we found coating SCC significantly increased proliferation, migration, and invasion of HDFa. At the molecular level, we demonstrated that SCC regulated HDFa invasion through Akt and MAPK signaling. In addition, coating SCC stimulated HDFa to increase gene expressions involved in ECM production and remodeling, including Col-Iα1, Col-IIIα1, elastin, Has 2, and MMP-1. It is suggested that the stiffness and composition of ECM is involved in regulating cell behaviors of fibroblast, including morphology, proliferation, differentiation, and migration [31,32]. Xie et al. demonstrated a lower proliferative rate of human mesenchymal stem cells on collagen with stiff fibers than that with soft fibers [33]. In addition, Stylianou et al. reported that an increase of collagen concentration elevated fiber density, stiffness, and contributed to an increase in cell spreading of fibroblasts [34]. In our study, we found the proliferation of HDFa was decreased by coating SCC at high concentrations, whereas the migration and invasion ability was increased, maybe due to the substrate stiffening.

Numerous studies have suggested the effect of tilapia type I collagen on the modulation of fibroblast proliferation and function as well as wound healing. Song et al. demonstrated that collagen from tilapia skin stimulated L929 fibroblast proliferation [26]. Electrospun collagen nanofibers were developed from tilapia skin and were shown to stimulate adhesion, proliferation, migration, and epidermal differentiation in human keratinocytes, as well as to facilitate wound healing in rats [11,12]. Other studies also reported the in vivo wound accelerating effect of tilapia type I collagen-derived peptide and tilapia type I collagen incorporated with other polymers [11,35,36], but the molecular mechanism of action remains unclear. Here we showed coating TSC significantly promoted proliferation, migration, and invasion of HDFa, in agreement with results of previous studies. The mechanism by which TSC promoted the invasion of HDFa was similar to that of SCC. Notably, TSC dramatically upregulated MMP-1 in HDFa, compared to coating SCC. MMP-1 is shown to cleave types I, II, and II collagen, and type I is the preferential substrate [37]. Coating TSC (type I collagen) may stimulate the upregulation of MMP-1 in HDFa to further ECM degradation during tissue repair. Moreover, our in vivo study showed that the dorsal wound administration of SCC or TSC facilitated the wound healing process in mice, with reduced inflammation, increased fibroblasts proliferation, ECM deposition, and re-epithelialization. The application of both SCC and TSC enhanced elastin expression at day 5 to day 10, as well as the deposition of collagen in the wound of mouse skin, indicating their ability to recover the function of wounded skin [38].

A number of growth factors, cytokines, and chemokines, such as VEGF, bFGF (also known as FGF-2), TGF-β, and CTGF, have been reported to regulate wound healing by targeting various cell types including endothelial cells, keratinocytes, and fibroblasts. They form a complex signaling network to influence cellular behaviors and play multiple roles during the healing process [39]. Chen et al. reported that the application of type I collagen from tilapia skin on dorsal wounds of SD rats accelerated wound healing by reducing inflammatory infiltration, promoting fibroblasts proliferation, collagen synthesis, and re-epithelialization via upregulation of EGF, FGF, and CD31 in the skin tissue [10]. In rabbits with burn wounds applied with tilapia skin, collagen-derived peptides, combined with chitosan, demonstrated healing by promoting re-epithelialization, collagen fiber deposition, and the upregulation of FGF2 and VEGF in the skin tissue [40]. In our current study, we observed that the topical application of SCC and TSC significantly increased the levels of the above four growth factors in the skin wound, with a clearly different expressing pattern. TSC application resulted in abundant growth factor expression, mostly in the epidermis (rich in keratinocytes) and partially in the dermis (rich in fibroblasts). In contrast, a high expression of growth factors was found in the dermis, dermal, and subcutaneous white adipose tissue (consisting of preadipocytes and adipocytes) following SCC administration, and also in the epidermal layer. It is known that collagens and their interaction with other ECM components such as elastin, laminin, fibronectin, proteoglycans, and glycosaminoglycans form 3D fibrous networks that influence cell growth, attachment, migration, and differentiation [41,42]. The differences in amino acid composition, sequence, covalent cross-links, structural assemblies between type I (TSC) and type II (SCC) collagen may influence their structural and biological features by interacting with different ECM constituents, cellular receptors, growth factors, and cytokines, thus leading to create individual proper environments for adhesion, proliferation, and migration of various cell types participating in wound healing. It has been suggested that type I collagen forms super-twisted microfibrils and links to neighboring microfibrils, creating the quasi-hexagonal crystalline structure [43]. Type II collagen displayed the conformation of well-ordered cross-linking of nonhelical telopeptides. The wide cross-linking between type II collagen fibrils seems to be more stable than type I collagen [44]. The distinct structural features of type I and type II collagen may provide different biological properties for the cellular behaviors in wound healing. In fact, the different nature of surface morphology and topography is found to influence a wide range of behaviors in various cells [45,46]. However, this hypothesis requires further investigation.

The major challenge of the present study is the different conditions of collagen preparation for SCC and TSC. SCC was prepared by pepsin digestion that resulted in missing telopeptide regions of tropocollagen, but not in the case of TSC. Studies have suggested telopeptides are critical for the assembly and stabilization of collagen fibrils [47,48]. In future work, we will prepare pepsin-solubilized TSC and compare it with SCC to identify specific cell and molecular target for accelerating wound healing. 

## 4. Materials and Methods 

### 4.1. Extraction and Coating of Collagen

The SCC and TSC were prepared with a slightly modified method of Liang et al. [17] and Li et al. [19]. The cartilage was washed, chopped, and stirred in 0.1 M NaOH solution and 0.5 M ethylenediaminetetraacetic acid (EDTA) (pH 7.4) at 4 °C for 1 day, respectively. The cartilage samples were then soaked in 0.01 M HCl solution including 0.1% pepsin (*w*/*v*) at 4 °C for 12 h with continuous stirring followed by filtration through 5B filter paper. The supernatant was salted-out by adding NaCl (final concentration of 2 M) at 4 °C and centrifuged at 2500× *g* for 30 min, then we collected the precipitate which was collagen. The cartilage tissue on the filter paper was repeatedly extracted according to the above method until it no longer contained residue. For preparing of TSC, tilapia skin was stirred in 0.1 M NaOH at 4 °C for 60 min and then was immersed in 0.5 M acetic acid with a solid/liquid ratio of 1:50 (*w*/*v*) for 24 h. After centrifugation, the supernatant of tilapia skin was salted-out as described above. Collagen samples were dialyzed with deionized water in a MWCO 20 KDa dialysis membrane at 4 °C for five days then freeze-dried to obtain SCC and TSC for subsequent use. In the in vitro study, the indicated concentration of collagen was added in the culture dishes or well until the dish contained no water; then the pre-coated culture dishes or well could be used.

### 4.2. Characterization of SCC and TSC

The amino acid content of the collagen was determined using high-performance liquid chromatography (HPLC) (Agilent 1260). The denaturation temperature of the collagen was measured with differential scanning calorimetry (DSC) (200 F3, NETZSCH, Selb, Germany). The surface morphology of the collagen was determined with scanning electron microscopy (S3000N, HITACHI Ltd., Tokyo, Japan). For sodium-dodecyl-sulfate gel electrophoresis, collagen samples were dissolved in 0.5 M acetic acid. Electrophoresis was performed on an 8% resolving gel and 5% stacking gel. Proteins were stained with Coomassie brilliant blue R-250, then destained using a solution containing water, methanol, and acetic acid (13:5:2, *v*/*v*/*v*).

### 4.3. Cell Culture

Mouse embryo fibroblast NIH-3T3 cells and human keratinocytes HaCaT cells were purchased from American Type Culture Collection (Rockville, MD, USA) and grown in DMEM including 2 mM glutamine (Gibco BRL, Grand Island, NY, USA), 10% (*v*/*v*) fetal bovine serum, and 1% penicillin/streptomycin (10,000 units of penicillin/mL and 10 mg streptomycin/mL). The human dermal fibroblast adult cell (HDFa cells; a kind gift from Dr. Hsing-Chun Kuo) were cultured in fibroblast medium (FM) (ScienCell, no. 2301) supplemented with 2% (*v*/*v*) fetal bovine serum, 1% penicillin/streptomycin, and 1% fibroblast growth supplement. These three cells all incubated at 37 °C in a 5% CO_2_ humidified atmosphere.

### 4.4. Trypan Blue Assay

HaCaT cells, NIH-3T3 cells, and HDFa cells were seeded on SCC- or TSC-coated 24-well plates at a density of 1 × 10^5^ cells/well, 2.5 × 10^4^ cells/well, and 2.5 × 10^4^ cells/well, respectively. After being incubated at 37 °C in a 5% CO_2_ humidified atmosphere for 36 h, cells were washed with sterile PBS, treated with 0.25% trypsin–EDTA, and harvested. Cells were stained with 0.4% trypan blue and then counted under a light microscope.

### 4.5. Cell Invasion Assay

To investigate the effect of SCC and TSC on cell invasion in HDFa, the insert of transwell chambers (Corning Inc., Corning, NY, USA) were pre-coated with indicating concentration of SCC or TSC. HDFa (1 × 10^5^ cells/mL) in 100 µL serum-free medium were added to the upper compartment, and the lower chamber was added 600 µL FM containing 2% FBS. Then the cells were incubated for 16 h at 37 °C with 5% CO_2_. To observe whether MAPKs and Akt signaling were involved in TSC and SCC-mediated HDFa invasion, cells were placed on the insert which was pre-coated with indicating concentration of SCC or TSC for 1 h, and then inhibitors were added and incubated for a further 15 h. The inserts were fixed in 4% paraformaldehyde for 5 min. The cotton swab was used to remove the non-invaded cells in the upper compartment and stained with 0.1 µg/mL DAPI for 30 min. Under the microscope, the cells were counted in five random sights.

### 4.6. Scratch Wound Healing Assay

To investigate the wound healing effect of SCC and TSC on HDFa, the cells were placed in indicating concentration SCC-coated or TSC-coated 12-well plates at a density of 1.5 × 10^6^ cells/well for 12 h. After cells formed a confluent monolayer, they were scratched using a sterile pipette tip to create a wound and washed by PBS to remove cell debris, and then incubated with fresh medium for 12 h. Images of the migrated cells were taken with an inverted microscope (Olympus) at 0, 4, 8, and 12 h. The wound area was quantified by Cellsens software in each group. Wound healing closer (%) = (original size of the wound area – wound area)/original size of the wound area × 100%.

### 4.7. Real-Time Polymerase Chain Reaction (qRT-PCR)

Total RNA was isolated from the HDFa cells using Quick-RNA™ MiniPrep kit (ZYMO RESEARCH Inc, Irvine CA, USA) following the manufacturer’s protocol. The gene expression of the cell migration marker (N-cadherin, Snail, and MMP1) and ECM deposition (has2, collagen Iα1, collagen IIIα1, and elastin) in HDFa was detected using real-time PCR. Glyceraldehyde 3-phosphate dehydrogenase (GAPDH) was used as an internal control. Primers for gene analysis are listed in Table S4. RNA was reverse transcribed using SuperScript™ III First-Strand Synthesis SuperMix for qRTPCR in accordance with the manufacturer’s instructions. Sample cDNA was subjected to qPCR reactions with an StepOnePlus™ real time system (Applied Biosystems, Foster City, CA, USA) using a fast protocol with universal cycling conditions (5 min at 95 °C, followed by 40 cycles of 20 s at 94 °C, 30 s at 60 °C, and 30 s at 72 °C). Real-time PCR was performed in a final reaction volume of 25 µL containing 2 µL; cDNA, 12.5 µL Fast SYBR^TM^ green master mix (Roche, Rotkreuz, Switzerland), 0.4 µL F primer, and 0.4 µL R primer. Primers were used at a final concentration of 10 mM in the reaction mix. Data was analyzed using the Applied Biosystems 7500 software suite with differential expression determined by the Comparative CT method (^ΔΔ^CT).

### 4.8. Excisional Wound Model

Forty male C57BL/6J mice (8–10 weeks old) were purchased from the BioLASCO Taiwan Co., Ltd. (Taipei, Taiwan). The mice were housed and maintained at controlled conditions with a 50% relative humidity, 12 h light/ dark cycle, and 22 ± 1 °C. All animals were free access to water and a standard diet. The animal study was approved by the Institutional Animal Care and Use Committee of Chiayi Chang Gung Memorial Hospital (Affidavit of Approval of Animal Use Protocol No. 2018062505; date of approval, 08 December 2019). After one-week acclimation, mice were randomly divided into the following five groups (n = 8): Control, SCC, and TSC (100 or 500 μg/cm^2^). Mice were anesthetized by intramuscular injection of Zoletil 50 (Virbac Taiwan Co., Ltd, Taipei, Taiwan) mixed with Rompun (Bayer, Leverkusen, Germany) (4:1), and two 8-mm wounds were made in their dorsal skin by biopsy punch. The wound area was treated with the indicated concentration of SCC or TSC then covered with the 3M™ Tegaderm™, and the dressings were changed every two days. The area of the skin wound was calculated by daily digital photography. The animals were sacrificed at 5 and 10 days after surgery via an overdose of isoflurane, and the skin tissues were collected and fixed in formalin, embedded in paraffin, and sectioned for histopathological examination and immunohistochemistry.

### 4.9. Histopathological Examination and Immunohistochemistry

The wounded skin samples were paraffin-embedded and cut into 3-μm-thick sections, stained with hematoxylin and eosin (H&E) or Masson’s trichrome stain and examined under a light microscopy (Olympus, Tokyo, Japan). For immunohistochemical staining, the wound skin sections were heated in a retrieval buffer (citrate buffer, pH 6.0) for 15 min to increase immunoreactivity. The sections were then incubated in a peroxidase-blocking solution for 10 min. The ECM deposition and growth factor in wounded skin were detected by incubating primary elastin, VEGF, bFGF, TGF-β, and CTGF antibodies for 1 h and subsequently polydetector HRP for 30 min. The immunostaining was performed by using a Mouse/Rabbit PolyDetector HRP/DAB Detection kit (BioSB, Santa Barbara, CA, USA). The ImageJ 1.46v software (US National Institutes of Health, Bethesda, United States) was used to quantify the immunostaining in each section.

### 4.10. Statistical Analysis

Statistical Analysis System (SAS) v 9.1 software was used for statistical analysis. Results were presented as means ± SE. The significant differences among the groups were performed using one-way ANOVA test and further with post-hoc Duncan’s multiple-range test. A *p*-value < 0.05 was considered statistically significant.

## 5. Conclusions

Our results demonstrated the potential effect of SCC, the type II collagen derived from the cartilage of *Acipenser baerii*, on fibroblast proliferation, migration, and invasion, as well as on ECM synthesis. Additionally, SCC application accelerated wound healing in the mouse skin, demonstrating a cell type-specific target compared to TSC. This study revealed the potential application of SCC on skin wound healing. Owing to the different cell types targeted, a combination of SCC and TSC could synergistically enhance wound healing.

## Supplementary Materials

The following are available online at https://www.mdpi.com/1660-3397/18/10/511/s1, Figure S1: SDS-PAGE pattern and SEM images of collagen from the cartilage of *Acipenser baerii* (SCC), Table S1: Proximate compositions of cartilage from *Acipenser baerii*, Table S2: Collagen yield of cartilage from *Acipenser baerii*, Table S3: Amino acid profile of SCC and tilapia skin collagen (TSC). Table S4, List of primers used for qRT-PCR analysis.
