# Supplementary material for: Type II Collagen from Cartilage of Acipenser baerii Promotes Wound Healing in Human Dermal Fibroblasts and in Mouse Skin
Source: Mar Drugs. 2020 Oct 11;18(10):511. doi: 10.3390/md18100511 (PMC7601416; doi:10.3390/md18100511)
Supplement: Supplementary file 1 [file marinedrugs-18-00511-s001.pdf]

Supplementary Materials

Type II Collagen from Cartilage of *Acipenser baerii* Promotes Wound Healing in Human Dermal Fibroblasts and in Mouse Skin

Ching-Shu Lai [1], Chun-Wei Tu [1], Hsing-Chun Kuo [2], Pei-Pei Sun [1] and Mei-Ling Tsai [1,*]

[1] Department of Seafood Science, National Kaohsiung University of Science and Technology, Kaohsiung 811, Taiwan; chlai@nkust.edu.tw (C.-S.L.); 1061534108@nkust.edu.tw (C.-W.T.); ppsun@nkust.edu.tw (P.-P.S.)

[2] Department of Nursing, Division of Basic Medical Sciences, Chang Gung University of Science and Technology, Chiayi, 61363, Taiwan; guscsi@gmail.com

* Correspondence: mltsai@nkust.edu.tw; Tel.: +886-7-361-7141 Ext 23623

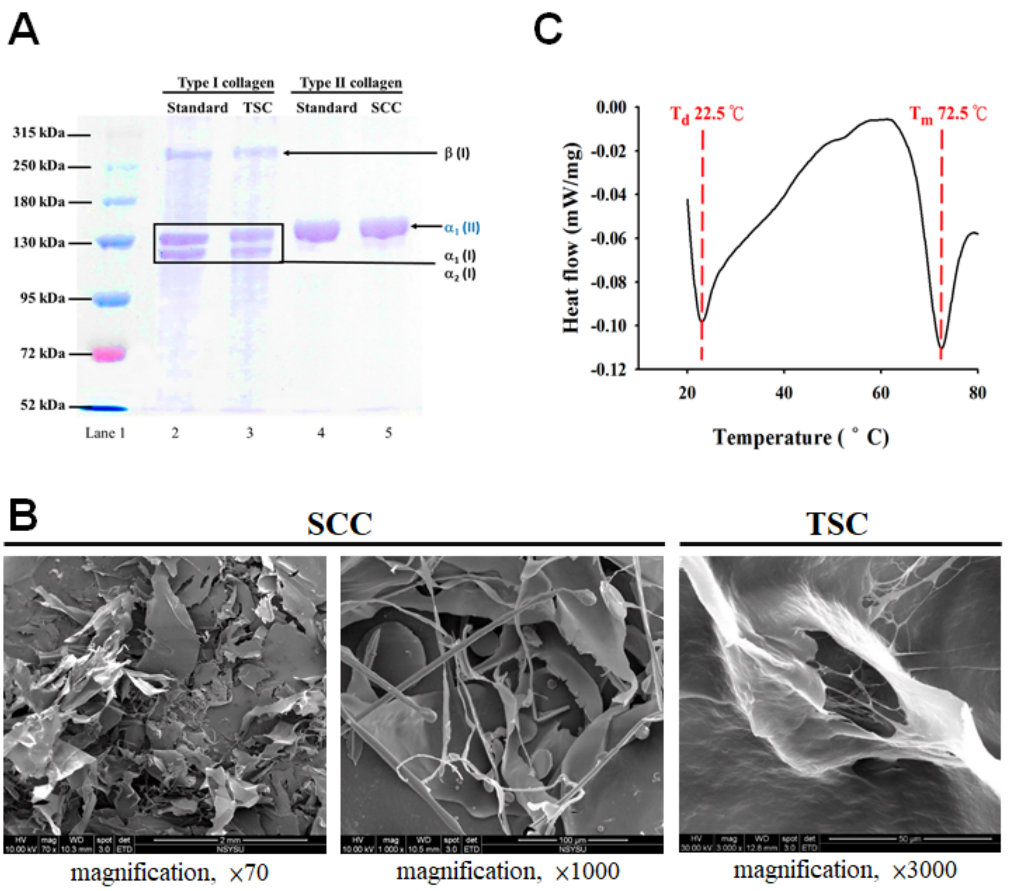

Figure S1. SDS-PAGE pattern and SEM images of collagen from the cartilage of *Acipenser baerii* (SCC). **(A)** Lane 1, molecular weight marker; lane 2, type I collagen standard from rat tail; lane 3, TSC; lane 4, type II collagen standard from chicken sternal cartilage and lane 5, SCC. **(B)** SEM images of lyophilized SCC and TSC. **(C)** DSC thermograms of SCC. X axis, temperature in °C; Y axis, heat flow.

Table S1. Proximate compositions of cartilage from *Acipenser baerii*

Composition	Dry weight (%)
Crude protein[a]	61.7 ± 0.66
Crude fat	6.39 ± 1.87
Ash	9.32 ± 1.21
Carbohydrates	21.7 ± 1.04
Total	99.15

All values represent the mean of three replicates ± standard deviation.

[a] % protein =%total nitrogen × 5.95

[b] % Carbohydrate calculated by difference

Table S2. Collagen yield of cartilage from *Acipenser baerii*

Characteristics	
Hydroxyproline (mg/g)	28.71 ± 0.48
Collagen (%)[a]	28.77 ± 0.48
Yield (%)[b]	20.11 ± 0.44
Extraction (%)[c]	69.92 ± 1.53

[a] The collagen content was obtained from hydroxyproline content with a factor of 10.02, which was calculated from the present result of amino acid profile (Table S3).

[b] Yield (%) = weight of lyophilized sample (g) / total solid weight of initial cartilage (g) × 100%

[c] Extraction (%) = yield /collagen × 100%

Table S3. Amino acid profile of SCC and tilapia skin collagen (TSC)

Amino acid	SCC	TSC
Gly	345.30	351.84
Pro	113.63	121.33
Hyp	99.79	98.60
Glu	92.24	64.09
Ala	89.38	128.01
Arg[a]	45.64	51.95
Asp	44.64	36.45
Ser	36.39	32.06
Leu[a]	32.22	19.88
Phe[a]	25.43	15.43
Thr[a]	22.20	24.76
Val[a]	16.40	15.31
Lys[a]	13.54	22.78
Ile[a]	11.69	7.33
Met[a]	7.33	8.18
Tyr	3.18	0.91
Cys	1.00	1.08
His[a]	-	-
Total	1000.00	1000.00
Imino acid[b]	213.42	219.93

[a] Essential amino acids

[b] Imino acids, include proline and hydroxyproline

Table S4. List of primers used for qRT-PCR analysis

Gene (human)	Primer sequence
Snail	F-TCGGAAGCCTAACTACAGCGA<br>R-AGATGAGCATTGGCAGCGAG
MMP1	F-AAAATTACACGCCAGATTTGCC<br>R-GGTGTGACATTACTCCAGAGTTG
N-cadherin	F-TCAGGCGTCTGTAGAGGCTT<br>R-ATGCACATCCTTCGATAAGACTG
Has2	F-CTCTTTTGGACTGTATGGTGCC<br>R-AGGGTAGGTTAGCCTTTTCACA
Collagen Iα1	F-GAGGGCCAAGACGAAGACATC<br>R-CAGATCACGTCATCGCACAAC
Collagen IIIα1	F-GGAGCTGGCTACTTCTCGC<br>R-GGGAACATCCTCCTTCAACAG
Elastin	F-GCAGGAGTTAAGCCCAAGG<br>R-TGTAGGGCAGTCCATAGCCA
GAPDH	F-TGTGGGCATCAATGGATTTGG<br>R-ACACCATGTATTCCGGGTCAAT
